# The Bioaccessibility and Bioavailability of Pentachlorophenol in Five Animal-Derived Foods Measured by Simulated Gastrointestinal Digestion

**DOI:** 10.3390/foods13081254

**Published:** 2024-04-19

**Authors:** Quan Zhou, Huiming Chen, Liangliang Li, Yongning Wu, Xingfen Yang, Aimin Jiang, Weiliang Wu

**Affiliations:** 1 The National Center for Precision Machining and Safety of Livestock and Poultry Products Joint Engineering Research Center, College of Food Science, South China Agricultural University, Guangzhou 510642, China; sonveri@163.com; 2 Guangdong Provincial Key Laboratory of Tropical Disease Research, Guangdong-Hongkong-Macao Joint Laboratory for Contaminants Exposure and Health, Food Safety and Health Research Center, School of Public Health, Southern Medical University, Guangzhou 510515, China; 13516509868@163.com (H.C.); fexjzhen@163.com (L.L.); yangalice79@smu.edu.cn (X.Y.); 3 Key Laboratory of Food Safety Risk Assessment, National Center for Food Safety Risk Assessment, Ministry of Health, Beijing 100021, China; wuyongning@cfsa.net.cn

**Keywords:** pentachlorophenol, in vitro digestive model, bioaccessibility, bioavailability, culinary treatment, food matrix, digestion phase, exposure risk

## Abstract

Pentachlorophenol (PCP) is a ubiquitous emerging persistent organic pollutant detected in the environment and foodstuffs. Despite the dietary intake of PCP being performed using surveillance data, the assessment does not consider the bioaccessibility and bioavailability of PCP. Pork, beef, pork liver, chicken and freshwater fish *Ctenopharyngodon Idella*-fortified by three levels of PCP were processed by RIVM and the Caco-2 cell model after steaming, boiling and pan-frying, and PCP in foods and digestive juices were detected using isotope dilution–UPLC-MS/MS. The culinary treatment and food matrix were significantly influenced (*p* < 0.05) in terms of the bioaccessibility and bioavailability of PCP. Pan-frying was a significant factor (*p* < 0.05) influencing the digestion and absorption of PCP in foods, with the following bioaccessibility: pork (81.37–90.36%), beef (72.09–83.63%), pork liver (69.11–78.07%), chicken (63.43–75.52%) and freshwater fish (60.27–72.14%). The bioavailability was as follows: pork (49.39–63.41%), beef (40.32–53.43%), pork liver (33.63–47.11%), chicken (30.63–40.83%) and freshwater fish (17.14–27.09%). Pork and beef with higher fat content were a key factor in facilitating the notable PCP bioaccessibility and bioavailability (*p* < 0.05). Further, the exposure of PCP to the population was significantly reduced by 42.70–98.46% after the consideration of bioaccessibility and bioavailability, with no potential health risk. It can improve the accuracy of risk assessment for PCP.

## 1. Introduction

Pentachlorophenol (PCP) and its salts are synthetic polychlorinated organic chemicals [1]. Owing to its excellent insecticidal and herbicidal properties, PCP has been widely used as an insecticide, fungicide, algaecide, herbicide, water clarifier, disinfectant, and wood preservative in agricultural and industrial fields since the 1930s [2,3]. In particular, PCP was widely used as a molluscicide in Southern China during the 1960s–90s to prevent the then-prevalent schistosomiasis [4]. As a fat-soluble persistent organic pollutant (POP), PCP is bioaccumulative and environmentally persistent and can undergo long-range migration, thereby contributing to global environmental pollution. Currently, PCP can be detected in several environmental media, including water, sediment, soil, and air, as well as in plants and animals [4,5,6]. Additionally, certain regions have been identified to be affected by severe PCP pollution. Therefore, the Stockholm Convention has listed PCP as a POP slated for elimination from both production and use [7].

This is because PCP is not easily biodegraded in the environment and has the propensity to accumulate in food products, causing contamination [7,8,9,10]. Basheer et al. detected PCP in seafoods, with its concentration ranging from 37.7 μg/kg ww (marine fish) to 146 µg/kg ww (marine crab) [11]. In China, a survey of aquatic species was performed to monitor the contamination of aquatic products by the historical use of PCP as a molluscicidal agent, and the PCP level was ranged from <0.5 to 61 μg/kg ww (common carp) [12]. A more extensive survey recently reported that the highest average values of PCP were found in livestock (beef and lamb), freshwater fish, and poultry, at 11.7, 11.6 and 9.9 μg/kg ww, respectively [13]. Additionally, among 12 samples of food packaging composed of recycled paper/cardboard, PCP was detected in 5 samples with a concentration of 54–110 μg/kg, whereas in 16 products composed of original paper, PCP was not detected [14]. According to the literature cited above, the occurrence of PCP in most food samples is far beyond the limit of 10 μg/kg in Europe [15]; moreover, exposure to PCP occurs not only through food but also via food contact materials. The results of risk assessment reveal that dietary exposure is an important pathway for PCP exposure, which results in a considerable health risk for general residents and a significant risk for the preschool-aged children [13]. Animal-derived foods with high fat content are considered as a major contributor [10,16,17].

Long-term exposure to PCP through dietary exposure and its accumulation in the body can lead to toxic effects and may cause prolonged and more serious health consequences. Toxicological literature has reported that PCP and its oxidative metabolites have relatively high electrophilicity and can easily bind to DNA and proteins, affecting their activity. PCP is thus considered an environmental mutagen. Furthermore, PCP exhibits reproductive/developmental toxicity, immunotoxicity, endocrine-disrupting toxicity, and hematologic toxicity [18,19]. Even more alarmingly, based on results from cohort and case–control studies, the U.S. Department of Health and Social Services, U.S. Environmental Protection Agency (U.S. EPA), and International Agency for Research on Cancer (IARC) have concluded that PCP exposure is closely associated with Non-Hodgkin’s lymphoma development [18,19]. IARC considers that the current data are sufficient to establish a causal relationship; therefore, they revised the classification of PCP from group 2B (possibly carcinogenic to humans) to group 1 (carcinogenic to humans) [19]. A case–control study published after the IARC report demonstrated that PCP exposure was associated with thyroid cancer in the general population [20].

Therefore, international organizations and federal governments have established standards for water and food that may come in contact with PCP. For instance, the World Health Organization (WHO) developed a temporary guideline to limit the PCP concentration in potable water to less than 9 μg/L [21], U.S. EPA mandates the maximum contamination level of PCP in potable water to be 1 μg/L [22], and the Commission of European Union stipulates a default PCP limit of 10 μg/kg for all foods in Europe [15]. Water quality standard for fisheries (GB 11607) established by the Ministry of Ecology and Environment of China in 1989 mandates that the PCP concentration of fishery water should not exceed 10 μg/L. China has gradually restricted the production and use of PCP since 1997 and issued the “List of Prohibited Veterinary Medicines and Their Derivatives for Food Animals” and “Blacklist of Illegal Non-Food Substances in Food (Part V)” in 2002 and 2011, respectively, to safeguard the inclusion of PCP in food.

In order to explore the hazardous characterization of the PCP exposure of the population, dietary exposure assessment of PCP in food should be performed to determine its exposure characteristics. However, chemical contaminants do not exist as monomers in the food matrix. Their structure and physical and chemical properties, as well as the binding form of proteins, carbohydrates, fats, and other substances in food, affect the activities of digestive enzymes in saliva, gastric juice, and small intestinal juice, which influence the enzymatic breakdown of bound pollutants and the rate at which chemical pollutants are transported across the intestinal barrier into blood circulation [23]. Thus, conventional dietary exposure assessment does not take digestion and absorption factors in the human body into consideration, possibly overestimating or underestimating population exposure levels and toxicity [24,25,26,27,28,29,30]. Clarifying the biological efficiency of pollutant digestion, absorption, and degradation in the gastrointestinal tract can help optimize the exposure assessment technology and improve its accuracy.

Recently, the health risk of dietary exposure to PCP was evaluated by deterministic [1] and probabilistic assessment [13], respectively. However, the bioaccessibility and bioavailability of PCP have not been considered in these two researches, and to the best of our knowledge, no report has studied the effects of the food matrix and cooking process on the bioaccessibility and bioavailability of PCP in foods [31]. Therefore, this study used Rijksinstituut voor Volksgezondheid en Milieu (RIVM) as an in vitro digestion model to systematically estimate the bioaccessibility of PCP in animal-derived foods. By simulating the digestive tract environment, we firstly investigated the effects of three different cooking methods, namely steaming, boiling, and pan-frying, and different digestive stages on PCP bioaccessibility in five animal-derived foods (beef, pork, pork liver, chicken, and freshwater fish *Ctenopharyngodon idella*). PCP bioavailability in foods of animal origin was further examined using a Caco-2 cell model [32]. This study aimed to provide a more comprehensive evaluation of PCP exposure caused by the consumption of animal-derived foods and a theoretical basis for the health risks associated with it.

## 2. Materials and Methods

### 2.1. Sample Collection

The five animal-derived foods, beef, pork, pork liver, chicken, freshwater fish (5 kg each), and peanut cooking oil (500 mL), were purchased from Jiajialian Supermarket in Baiyun District, Guangzhou City, China. The food samples were rinsed and excess water from the surface was removed using a clean gauze. After slicing the samples on a plastic chopping board, they were blended in a meat grinder until thoroughly minced. They were then placed in transparent food containers and stored in a refrigerator at −18 °C.

### 2.2. Preparation of Spiked Samples and Culinary Treatments

According to our previous surveillance results [1], three scenarios were arranged to simulate the low (100 μg/kg), medium (600 μg/kg) and high (1200 μg/kg) contamination in animal-derived foods. The fortified samples of PCP were prepared, following the methods described by Shen et al. [30]. Briefly, 25 mg (±0.01 mg) PCP (Dr. Ehrenstorfer GmbH, Augsburg, Germany) was firstly dissolved into 12.5 mL methanol to achieve a PCP stock solution of 2000 μg/mL. PCP standard working solutions of three levels were further diluted for application using deionized water. Then, 500 g of the sample were taken for each food type. After homogenizing the samples with a high-speed blender, they were freeze-dried for 72 h and then crushed. Subsequently, PCP standard working solution was added to the samples separately to obtain food-spiked samples with the above-mentioned concentrations. The spiked samples were vortexed for 60 min and then sealed in glass bottles for incubation for 6 h at 40 °C. Finally, the samples were cooled to room temperature and placed in a refrigerator at 4 °C for 1 week before cooking. To test the PCP concentration in the spiked samples, 5.0 g of the samples were collected in triplicate.

Ultrapure water was added to 100 g of spiked samples in a 1:3 solid–liquid ratio to rehydrate the samples, which were then divided into three parts. The three samples were then cooked according to the cooking methods and conditions described in Table 1, and analysis was conducted to confirm no PCP could be quantified in peanut oil before it was used for cooking. After the collected samples were pre-processed, the concentration of PCP in the five food products after cooking with different methods was determined using isotope dilution-ultra-high performance liquid chromatography-triple quadrupole mass spectrometry (UPLC-MS/MS).

### 2.3. In Vitro Digestion Procedure

The process of in vitro gastrointestinal digestion was performed in a model with three cavities, including mouth, stomach, and small intestine, for the evaluation of PCP bioaccessibility followed by the procedure described by Oomen et al. [31] and Xu et al. [27] with minor modifications. The constituents used for each digestive compartment are provided in Table S1. Briefly, the food sample was firstly vortexed at 37 °C for 2 min after 6 mL saliva was added to a 5 g cooked sample for digestion. Subsequently, an aliquot of 13 mL synthetic gastric juice with pH of 1–2 was loaded into the digesta and incubated at 37 °C, 80 rpm for 2 h. Then, the chyme was digested at 37 °C, 80 rpm for another 2 h after the injection of 12 mL of duodenal juice (pH 7.8) and 6 mL of bile juice (pH 8.0). Finally, the process was completed by boiling the mixture for 30 s at 100 °C, followed by being centrifuged at 5000 rpm for 10 min for the separation of the supernatant of the digestive juice from the residues. Both 1 mol/L HCl and NaHCO_3_ were used to adjust the pH of the juice during the process of digestion. The filtration of the supernatant was performed using a 0.22-μm filter for in vitro adsorption using the Caco-2 mono-culture cell model and analytical determination using the ultra-high performance chromatographic method with triple-quadrupole mass spectrometry detection.

### 2.4. In Vitro Intestinal Model

#### 2.4.1. Model Building

The cell used for building the in vitro adsorption method was the Caco-2 cell line (ATCCP^®^P HTB-37TM), which was supplied by American Type Culture Collection (Manassas, VA, USA). The cell culture protocol was performed according to the procedure reported by the previous literature [32,33], with minor revisions. In brief, a complete medium, consisting of Eagle’s Minimum Essential Medium (EMEM, ATCC, Manassas, USA), 20% (*v*/*v*) heat-inactivated fetal bovine serum (FBS, ExCell Bio Inc., Shanghai, China) and 1% (*v*/*v*) penicillin/streptomycin (Gibco, Rockville, MD, USA), was employed to routinely culture Caco-2 cells (passages 19−30) at 37 °C in 5% CO_2_. After 8 days of cultivation, the cells were loaded into the upper apical of a TranswellP^®^P plate (24-well, clear-polyester membrane, 0.4 μm pore size, Corning Life Sciences, Lowell, MA, USA) for mono-culture with a seeding density of 5.0 × 10^4^ cells/cm^2^ in 0.2 mL complete EMEM, while the lower basolateral chamber had 0.6 mL complete EMEM.

#### 2.4.2. Model Verification

An in vitro intestinal model constructed using the Caco-2 cell line was verified by three indicators, including transmembrane resistance, alkaline phosphatase, and lucifer yellow permeability, according to the literature reported by Hubatsch et al. [34] and Pick et al. [35].

##### Transmembrane Resistance

The indicator of transepithelial electrical resistance (TEER) was measured by epithelial volt–ohmmeter (EVOM2, World Precision Instruments, Sarasota, FL, USA) to monitor the differentiation process of gastrointestinal epithelial cells. The value of TEER above 250 Ω/cm^2^ indicated the monolayer model for in vitro adsorption testing had been established successfully. The TEER value of the model used in this work was in the range of 500–560 Ω/cm^2^, which was consistent with the results of over 500 Ω/cmP^2^ Preported by Chen et al. [36] and Shi et al. [37].

##### Alkaline Phosphatase

As the characteristic enzyme in Caco-2 cells, alkaline phosphatase (AKP) is mainly concentrated in the upper chamber after cell differentiation. After the cells were seeded in Transwell plates for 21 days, culture media on the apical chamber (AP) and basolateral chamber (BL) sides were taken and the concentration of AKP was tested using an AKP kit (Abcam, Cambridge, UK). This experiment determined the nature of cell polarity, where polarity in the AP side should be higher than that in the BL side.

##### Lucifer Yellow Permeability Assay

Hank’s Balanced Salt Solution (HBSS) was used to prepare Lucifer yellow standard solutions of various concentrations: 0.005, 0.01, 0.025, 0.05, 0.1, and 0.25 μg/mL. Fluorescence intensity was measured using an enzyme marker (excitation wavelength 427 nm, emission wavelength 536 nm). Using fluorescence intensity as the vertical coordinate and concentration as the horizontal coordinate, regression analysis was performed and standard curves were drawn. Thereafter, the culture media from three wells were removed. The AP and BL sides were rinsed twice with prewarmed HBSS. After discarding the remaining HBSS, 0.5 mL of Lucifer yellow (20 μg/mL) was added on the AP side and 1.5 mL of HBSS was added on the BL side. The cells were incubated for 2 h at 37 °C and 5% CO_2_. Then, 100 μL samples were taken from the BL side at 60 and 120 min and equal volumes of HBSS blank were added. Fluorescence intensity was detected using a plate reader at an excitation wavelength of 427 nm and emission wavelength of 536 nm. The concentration of Lucifer yellow in the BL-side transporter and the Lucifer yellow permeability coefficient were calculated based on the standard curve and regression equation to verify the permeability of Caco-2 monolayer cells. A Papp value of less than 5.0 × 10^−7^ cm/S demonstrates adequate permeability and compactness of the cell monolayer.

(1) $$Papp=\frac{\frac{dQ}{dt}}{AC_{0}}$$

In the above formula, dQ/dt is the amount of fluorescent yellow translocation per unit time (μg/S), A is the effective membrane area (1.21 cm^2^), and C_0_ is the initial concentration of fluorescent yellow (20 μg/mL).

#### 2.4.3. Bioavailability Measurement

##### CCK-8 Experiment

Since both the simulated digestion medium and pollutants in the intestinal phase are toxic to Caco-2 cells to a certain extent, a CCK-8 experiment was performed to determine the appropriate experimental dosage of intestinal medium after digestion using the CCK-8 kit (Dojindo, Kumamoto, Japan) [27,36,37,38]. When cell viability was greater than 85%, a transport experiment was performed.

After cells reached confluency in the culture bottles, 0.25% trypsin was used for digestion and added at 100 μL/well into 96-well cell culture plates with a cell density of 1.25 × 10^5^/mL. The cells were incubated at 37 °C, relative humidity 90%, and 5% CO_2_ for 24 h. Digestion media in the intestinal phase in the in vitro digestion method and HBSS buffer were mixed in 1:0, 4:1, 2:1, 1:1, 1:2, 1:4, and 1:5 ratios. These mixtures (100 μL each) were added to 96-well plates, and 100 μL HBSS buffer was added to the blank. Each treatment had six replicates. After incubating the culture plates in the incubator for 24 h, 10 μL of CCK-8 solution was added to each well (1:10 dilution using culture media). Subsequently, the culture plates were incubated for 1–4 h, and the absorbance was measured using a plate reader at 450 nm [27]. Before starting the bioavailability evaluation of PCP, the juice obtained from the RIVM employed for PCP in vitro digestion was diluted according to the results of the CCK8 assay (Figure S1) by EMEM as follows:

(2) $$Cell\;viability\left(\%\right)=\frac{A_{s}-A_{b}}{A_{c}-A_{b}}\times 100\%$$

where A_s_ is the absorbance of experimental wells (containing cells, medium, CCK-8 solution, and small intestine digestive solution), A_c_ is the absorbance of control wells (containing cells, medium, and CCK-8 solution), and A_b_ is the absorbance of blank wells (containing only medium and CCK-8 solution).

##### Bioavailability Assay

An HBSS solution prewarmed to 37 °C was added to the Caco-2 cell monolayer after culturing for 21 days. The cell surface was rinsed twice and the HBSS solution was added again. After balancing for 15 min, the HBSS solution was discarded. Thereafter, a 0.5 mL digestive medium in the aqueous phase was added to the AP side and a 1.5 mL HBSS buffer was added to the BL side [28,32]. The plate was incubated for 4 h in a thermostatic incubator. The AP-side solution was collected. The Caco-2 cell monolayer was rinsed with a trypsin/EDTA mixture three times and then separated. Thereafter, a 0.5 mL MEM/EBSS NEAA replenishment solution was used to resuspend the cells. The cells were collected, and the PCP in AP test solutions and cells was determined using the isotope dilution–UPLC-MS/MS method.

### 2.5. Sample Pretreatment and Instrumental Analysis

#### 2.5.1. Pretreatment

The purification process for fresh samples, cook samples and digests was followed by the National Food Safety Standard GB 23200.92-2016 [39] with minor revisions. Two grams of the raw samples, cooked samples, 10 mL digestive juices or 1 mL cell culture solution were firstly pipetted into polypropylene centrifuge tubes with 200 µL of 1 ng/µL P^13^PCR_6_R-PCP, and were homogeneous extracted in 6 mL of 5% triethylamine acetonitrile aqueous solution for 2 min and ultrasonic-assisted extracted for 5 min. Subsequently, the supernatants were collected after 5 min of centrifugation at 3000 rpm. Finally, the extraction was repeated, and two extracts were combined to load into Oasis MAX solid phase extraction columns (Waters Corporation, Milford, MA, USA) for purification using 5 mL of 5% ammonia solution, 5 mL of methanol, and 5 mL of 2% formic acid methanol–water solution for sequential elution. After that, the eluents washed by 4 mL of 5% formic acid methanol were collected into tubes to concentrate to 1 mL under gentle nitrogen flow. The solutions were filtered using 0.22 µm polytetrafluoroethylene organic filters (Agilent Technologies, Inc., Santa Clara, CA, USA) for instrumental analysis.

#### 2.5.2. Instrumentation

Quantitative analysis was performed on an Acquity UPLC H-class (Waters Crop., Milford, MA, USA) couple to a Waters Xevo TQD operated in the multiple reaction monitoring (MRM) mode. Separation was achieved on a Waters Acquity BEH C18 column (100 mm × 2.1 mm i. d. × 1.7 µm), held at 30 °C. The mobile phase was supplied at a constant flow rate of 250 µL/min. An aliquot of 10 µL sample was injected into the injector, and chromatographic analysis was performed by programming a gradient elution procedure, as shown in Table S2. The mass spectrometer was operated with an ESI source with negative ion mode at −4.5 kV; resolution: unit resolution; electrospray voltage: 4500 V; ion source temperature: 550 °C; curtain gas pressure (CUR): 20.00 psi (N_2_); atomizing gas pressure: 35 psi (N_2_); and drying gas pressure: 45 psi (N_2_). Quantitative ion pairs: 262.7 > 262.7; qualitative ion pairs: 264.7 > 264.7, 266.7 > 266.7, and 268.7 > 268.7.

#### 2.5.3. Quality Control and Assurance

The protocols of quality control and assurance for the validation of reliability, accuracy and precision of the determination method were as follows: (1) the qualitative results should meet the requirement of the maximum permitted tolerances described in Commission 2002/657/EC; (2) the blank samples of each five types of foods spiked with three levels of PCP standard solutions were prepared for recovery determination (Table S3); (3) the analytical sequence was performed as follows: procedural blank, blank matrix, fortified blank matrix, and each batch of ten samples; (4) PCP standard solution (Agro-Environment Protection Institute, Ministry of Agriculture, Tianjin, China) was obtained for methodological verification, including accuracy and precision; (5) the relative standard deviation of the coefficient of variation was calculated in triplicate to ensure analysis precision; (6) the limit of quantification (LOQ) of the method was 1.0 μg/kg ww.

### 2.6. Bioaccessibility and Bioavailability Calculation

The in vitro digestion and adsorption determinations were conducted in triplicates. The bioaccessibility of PCP was calculated as follows (Equation (3)):

(3) $$\mathrm{Bioaccessibility}\;(\%)=\frac{C_{1}\times V_{1}}{C_{2}\times M}\times 100\%$$

where C_1_ is the level of PCP in gastrointestinal juice (mg/mL), V_1_ is the total volume of the simulated digestive fluid used in the in vitro digestion (mL), C_2_ is the fortified level of PCP in different food matrices (mg/kg), and M is the weight of the sample for digestion (kg).

The bioavailability of PCP was calculated using the following equation [27]:

(4) $$Absorption\;rate\left(\%\right)=\frac{C_{1}\times V_{2}-C_{3}\times V_{3}}{C_{1}\times V_{2}}\times 100\%$$

where C_1_ is the initial PCP concentration in intestinal juice before the in vitro absorption test (mg/mL), V_2_ is the volume of intestinal juice loaded into AP for the in vitro absorption test (mL), C_3_ is the PCP concentration in intestinal juice after the in vitro absorption test (mg/mL), and V_3_ is the volume of the solution collected from AP after the in vitro absorption test (mL).

The bioavailability of PCP was defined as the total quantity of PCP absorbed by the in vitro adsorption model, including transported and retention parts, divided by the level of PCP in food samples. It was calculated using the following equation:

(5) $$Bioavailability\left(\%\right)=Bioaccessibility\left(\%\right)\times Absorption\;rate(\%)$$

### 2.7. Exposure Assessment

The following deterministic approach (Equation (5)) was carried out to estimate the daily dietary exposure of PCP via five food categories in consideration of the bioaccessibility and bioavailability of PCP:

(6) $$BioAcc\;EDI=\frac{C_{2}\times W_{v}\times BAS}{BW}$$

where C_2_ is the fortified level of PCP in different food matrices (mg/kg ww), W_v_ is the amount of animal-derived food consumed daily (kg/d), BioAcc EDI is the estimated daily exposure to PCP corrected for bioaccessibility, BAS is the bioaccessibility of PCP, and BW is the average body weight (kg). The amount of animal-derived food consumed daily (Table S4) and the average body weight were acquired from the NAHS [40] as follows:

(7) $$BioAva\;EDI=\frac{C_{2}\times W_{v}\times BAV}{BW}$$

where C_2_ is the fortified level of PCP in different food matrices (mg/kg), W_v_ is the amount of animal-derived food consumed daily (kg/d), BioAva EDI is the estimated daily exposure to PCP corrected for bioavailability, BAV is the bioavailability of PCP, and BW is the average body weight (kg).

### 2.8. Statistical Analysis

The relative standard deviations (i.e., coefficient of variation) from triplicate measurements were calculated to ensure analytical precision and that no contamination occurred during the entire process of analysis. All data are presented as mean or mean ± relative standard deviation (RSD). The related data were processed using SPSS 21.0. A comparison between the groups was carried out using ANOVA and the correlation between groups was determined using Pearson and Spearman correlation analyses. *p* < 0.05 was considered a statistically significant difference.

## 3. Results and Discussion

### 3.1. Quality Evaluation of Sample-Spiking Procedures

The PCP baseline levels in the five types of animal-sourced foods used in the in vitro digestion and absorption experiments are shown in Table 2. Except for the PCP background concentration of pig liver, which was 1.23 ± 0.16 μg/kg ww, the concentrations of the other four types of foods were below the LOQ. Since the minimum spiked concentration of all the samples used in the in vitro digestion and absorption experiments was 100 μg/kg ww, the PCP background concentration in the samples did not significantly affect the subsequent bioaccessibility and bioavailability experiments.

Studies have shown that while performing bioaccessibility and bioavailability analysis using spiked food samples, adequate interactions of spiked compounds with food matrices are one of the key steps in preparing spiked samples [30,41,42]. To validate whether spiked samples can be used to evaluate PCP bioaccessibility and bioavailability, we compared the difference between PCP bioaccessibility and bioavailability for the five types of spiked or naturally contaminated foods according to the previous report [30]. As shown in Table 3, PCP bioaccessibility between the spiked samples and blank samples of steamed pork and fried beef were significantly different (*p* < 0.05), and PCP bioaccessibility between the spiked and naturally contaminated pork and beef cooked with other methods were not significantly different (*p* > 0.05).

Further, for the other three food matrices under the three cooking treatments, PCP bioaccessibilities between the spiked samples and blank samples were not significantly different (*p* > 0.05). PCP bioavailability was not significantly different in the food matrices of spiked and blank samples. These findings indicate that the bioaccessibility and bioavailability of spiked and naturally occurring PCP are not significantly different for the five food types, suggesting that spiked samples can accurately reflect the interactions between PCP and food matrices [30]. Therefore, this study continues to use spiked samples to discuss the effects of different cooking methods and digestion phases on PCP bioaccessibility and bioavailability in the five food matrices.

### 3.2. Effects of Culinary Treatments on PCP Bioaccessibility in the Five Foods

After steaming, boiling, and pan-frying the five food types, a digestive experiment was performed using in vitro digestive model systems, and the results are shown in Table 4, Table 5 and Table 6. The replicability and reproducibility of in vitro digestion experiments were satisfactory, and the relative standard deviation of triplicated experiments was less than 10%, meeting the requirements of the relevant standards [27,28,31]. As shown in Table 4, Table 5 and Table 6, after the salivary digestion of the five food matrices containing different concentration levels of PCP, only a small fraction of PCP was released into the salivary digestive fluid via the food matrix and the bioaccessibility was 4.93–35.03%. Since the salivary digestive fluid only had amylase and relatively small digestive effects on the five animal-sourced foods, PCP could not be completely released from the food matrices.

Compared with the oral phase, gastric digestion significantly increased PCP bioaccessibility (*p* < 0.05), which reached 14.14–81.57%. This is possibly because, under the influence of the low pH environment of gastric juice and proteases, protein in animal-sourced foods was digested, released, and combined with PCP in the digestive medium to significantly increase the bioaccessibility of PCP (*p* < 0.05). This suggests that the gastric phase is the main phase for PCP release from the food matrix. Compared with digestion in the gastric phase, digestion in the duodenal phase caused some PCP to continue to release in the digestive medium, with PCP bioaccessibility reaching 21.82–90.36%. Comparing the PCP bioaccessibility in the small intestine digestion with that in the gastric digestion, the bioaccessibility of all the samples significantly increased after intestinal digestion (*p* < 0.05), except for boiled pork containing 100 μg/kg PCP, boiled and pan-fried pork containing 600 μg/kg PCP, boiled pork liver containing 600 μg/kg PCP, and boiled freshwater fish containing 600 μg/kg PCP (*p* > 0.05).

Table 4, Table 5 and Table 6 show that the cooking methods influence PCP bioaccessibility in the five animal-derived foods. At low concentration (100 μg/kg ww), PCP bioaccessibility in the five fried food samples during the oral phase was significantly higher than that in the boiled food samples (*p* < 0.05). Although PCP bioaccessibility in the steamed samples during the oral phase was higher than that in the boiled food samples and lower than that in the fried food samples, it was not statistically significant (*p* > 0.05). During the gastric phase, PCP bioaccessibility in the five fried samples was significantly higher than in the steamed and boiled food samples (*p* < 0.05), and PCP bioaccessibility in steamed pork, pork liver, and chicken was significantly different than that in the boiled food samples (*p* < 0.05). However, PCP bioaccessibility in steamed beef and freshwater fish was not significantly different than that in the boiled food samples (*p* > 0.05). During the intestinal phase, PCP bioaccessibility in steamed beef was not significantly different from that in the boiled food samples (*p* > 0.05); however, the cooking methods significantly affected PCP bioaccessibility in the other four food types (*p* < 0.05).

At medium concentration (600 μg/kg ww), the cooking methods had a significant effect (*p* < 0.05) on the PCP bioaccessibility in pork during the oral phase. The PCP bioaccessibility of the remaining four food matrices was also significantly higher after pan-frying compared with steaming and boiling (*p* < 0.05), whereas steaming and boiling did not have a significant effect on PCP bioaccessibility for the remaining four food matrices (*p* > 0.05). For the gastric digestion stage, cooking methods had a significant effect on the PCP bioaccessibility of pork, beef, and chicken *(p* < 0.05); in pig liver and freshwater fish samples, only pan-frying had a significant effect on PCP bioaccessibility (*p* < 0.05). In the small intestine digestion stage, cooking methods significantly influenced (*p* < 0.05) PCP bioaccessibility in pork, beef, chicken, and freshwater fish. However, steaming and boiling had no significant effect on PCP bioaccessibility in pork liver during the gastric and intestinal phases, or freshwater fish in the gastric phase (*p* > 0.05).

At high concentration (1200 μg/kg ww), all three cooking methods significantly (*p* < 0.05) affected PCP bioaccessibility in pork, beef, and freshwater fish in all three digestive phases. Steaming and boiling had no significant effect on PCP bioaccessibility in pork liver in the oral and gastric phases (*p* > 0.05) and chicken in the oral phase (*p* > 0.05), but had a significant effect (*p* < 0.05) on PCP bioaccessibility in pork liver during the small intestinal phase (*p* < 0.05) and chicken in the gastric and small intestinal phases (*p* < 0.05). PCP bioaccessibility in the three digestive phases was significantly higher (*p* < 0.05) in the fried pork liver and chicken samples than in the steamed and boiled food samples.

Upon comparing these results to previous related results, it is evident that different cooking methods can significantly alter pollutant bioaccessibility in foods. Among these, cooking methods using oil, such as deep frying or pan-frying, can substantially increase the bioaccessibility of POPs in food [43,44]. This is mainly attributed to the weak polar nature of POPs, limiting their distribution in steamed and boiled foods, but during pan-frying, the use of cooking oil can increase the solubility of POPs, including PCP. Because of the fat-soluble nature of PCP, the pan-frying cooking method significantly increased its bioaccessibility in the five animal-sourced foods (*p* < 0.05).

In general, similar results and variation tendencies were obtained from five animal-derived foods spiked by three levels of PCP treated by three different cooking methods. Based on our previous surveillance data, the low-spiked concentration of 100 μg/kg ww in animal-derived foods in this study closely resembled the real environmental contamination situation, while medium- and high-fortified concentrations simulated the scenario of heavy contamination or the illegal use of PCP as a preservative in food. It is clearly demonstrated that culinary treatment should be considered as a more important impact factor for PCP bioaccessibility than the level of PCP contamination. However, although PCP bioaccessibility had no significant correlation with PCP content in animal-derived food, greater concern should also be given to the status of more PCP released into digestive juice from a high-contamination sample.

### 3.3. Influence of Food Matrices on the Bioaccessibility of PCP

Previous studies have found that the bioaccessibility of POPs is affected by the food matrix; the fat content in food is one of the main factors influencing it [41,42]. In addition, the difference between food types is also a factor affecting bioaccessibility. As shown in Table S5, the food matrix had a significant effect on PCP bioaccessibility during different digestive phases (*p* < 0.05).

For the pan-frying process, pork and beef samples had significantly higher PCP bioaccessibility than that in the other three food matrices (*p* < 0.05) during three digestive stages at low PCP concentration (100 μg/kg ww). Although the bioaccessibility in fried pork liver, chicken and freshwater fish had no significant difference at the oral phase (*p* > 0.05), significant PCP bioaccessibilities (*p* < 0.05) among these three food samples were observed during the gastric stage; moreover, PCP bioaccessibility in fried pork liver was notably different (*p* < 0.05) than that in fried chicken and freshwater fish samples at the intestinal phase. At medium (600 μg/kg ww) and high (1200 μg/kg ww) PCP levels, significant bioaccessibility (*p* < 0.05) was observed in five fried food matrices at the initial digestive phase. Fried pig and beef had significant PCP bioaccessibility (*p* < 0.05) at the continuous two digestive phases compared with the other three food matrices.

For the boiling cooking method, remarkable PCP bioaccessibility (*p* < 0.05) was found in boiled pork (11.63% and 13.83%) with low and medium PCP levels in comparison with the other four food samples at oral digestion, while PCP bioaccessibility in boiled pork and beef had significant difference (*p* < 0.05) with the other three food matrices at high PCP level. Similarly, pork and beef with low and high PCP concentration after boiling had significantly higher (*p* < 0.05) PCP bioaccessibility than that in the other three food samples during stomach and intestine digestion. Differently, PCP bioaccessibility in boiled pork, beef, and pork liver was notably higher (*p* < 0.05) than in boiled chicken and freshwater fish samples, while freshwater fish had the lowest PCP bioaccessibility (*p* < 0.05) after gastric and intestinal digestion.

After oral digestion, PCP bioaccessibility in steamed pork had a significant difference with that in the other four food types at low PCP level (*p* < 0.05), while there was no significance observed in PCP bioaccessibility among the other four food samples (*p* > 0.05); steamed pork, beef and pork liver had significant bioaccessibility (*p* < 0.05) compared with steamed chicken and freshwater fish at medium and high PCP levels. During gastric and intestinal digestion, significantly higher PCP bioaccessibility was also found in steamed pork and beef (*p* < 0.05); relatively lower PCP bioaccessibility (*p* < 0.05) was observed in steamed pork liver and chicken. Moreover, the difference in PCP bioaccessibility between steamed pork liver and chicken was significant (*p* < 0.05) at low and medium levels, but not statistically significant (*p* > 0.05) at high PCP level; steamed freshwater fish had the lowest PCP bioaccessibility (*p* < 0.05) at all three PCP levels.

In summary, pork and beef, treated by all the cooking methods and with high fat content (pork: 12.72 ± 1.41 g/100 g ww; beef: 8.12 ± 1.94 g/100 g ww), had the highest PCP bioaccessibility (47.92–90.36%) during the oral, gastric, and intestinal phases, and the PCP bioaccessibility of these two foods was significantly different from that of other foods (*p* < 0.05). Pork liver (5.14 ± 0.58 g/100 g ww) and chicken (3.79 ± 0.76 g/100 g ww) samples with slightly lower fat content had the second-highest PCP bioaccessibility, and there were significant differences (*p* < 0.05) in the bioaccessibility of PCP between the two food types at different phases of digestion, which were influenced by both the fat content and the cooking method. The PCP bioaccessibility of freshwater fish, which possess the lowest fat content (2.42 ± 0.62 g/100 g ww), was the lowest in all the digestive phases and was significantly lower than that in other food matrices in most cases (*p* < 0.05).

To some extent, fat content plays an important role in bioaccessibility. The POP polychlorinated biphenyls (PCBs) in animal-derived foods are more bioaccessible than PCBs in rice and cabbage [42]. Yu et al. [43] reported that the bioaccessibility of polybrominated diphenyl ethers (PBDEs) is proportional to the fat content of uncooked animal-sourced food, which is consistent with the findings of the present study. Furthermore, Wang et al. reported that the mean values of hexachlorocyclohexane (HCH) and dichlorodiphenyltrichloroethane (DDT) bioaccessibility in freshwater fish samples are 8.73% and 17.6%, respectively [44]; these are similar to the PCP bioaccessibility values for steamed and boiled food samples in this study.

### 3.4. In Vitro Bioavailability Modeling and Validation

Before evaluating PCP bioavailability in animal-sourced foods, it is essential to verify whether the in vitro Caco-2 cell monolayer model is successfully constructed for bioavailability. Caco-2 cells were seeded on a 24-well Transwell polyester membrane for differentiation, and the compactness and integrity of the Caco-2 cell monolayer model was observed after 21 days using a microscope. As shown in Figure S2A, the compactness and integrity of the Caco-2 cell model were satisfactory. The transmembrane resistance value of the Caco-2 cell model was significantly higher than 250 Ω-cm^2^ (Figure S2B). Additionally, AKP activity was greater on the AP side than on the BL side (Figure S3C). All these observations indicated that the Caco-2 cell model was successfully constructed. The Lucifer yellow permeability coefficient of (0.45 ± 0.15) × 10^−6^ also met the requirements of the relevant standards (Figure S3D). The comprehensive results from the four indices demonstrated that the in vitro bioavailability model was successfully constructed.

### 3.5. Effects of Cooking Methods on the Bioavailability of PCP in the Five Foods

**Figure 1:**
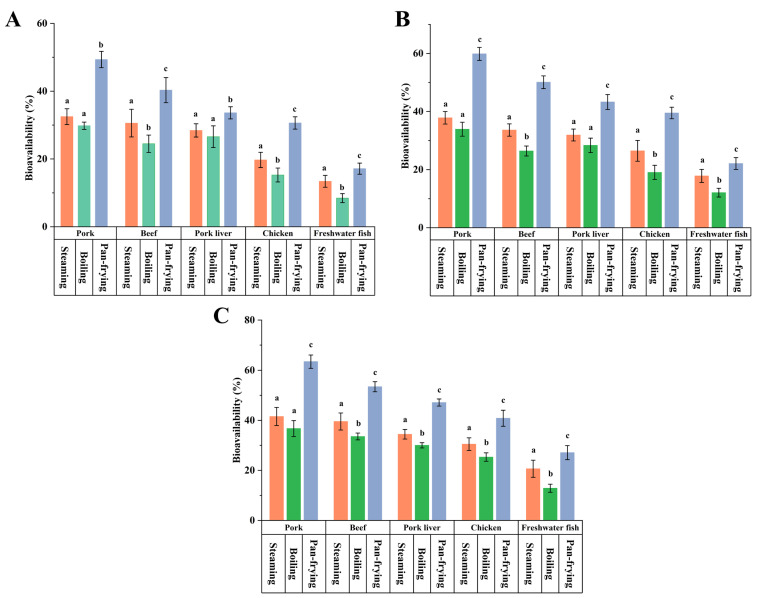
Effects of culinary treatments on the bioavailability of pentachlorophenol: (**A**) 100 μg/kg ww food samples; (**B**) 600 μg/kg ww food samples; (**C**) 1200 μg/kg ww food samples. Each food matrix treated by different cooking methods with the mean values of different lowercase letters are statistically significant (*p* < 0.05).

As shown in Figure 1, in food samples with three PCP-spiked concentrations, low, medium, and high, the bioavailability range of PCP was 12.86–63.41%; in all cases, the pan-frying process significantly increased the bioavailability of PCP in foods (*p* < 0.05) in the range of 17.14–63.41%. The bioavailability of PCP was below 40% in both steamed and boiled foods, indicating that only less than 40% of the PCP released in the digestive fluid during digestion is taken up by Caco-2 cells and enters the circulatory system, becoming an internal exposure dose that affects the target organs. The bioavailability of the PCP of steamed and boiled foods was significantly lower than that of pan-fried samples (*p* < 0.05). This is mainly attributed to the fact that the level of PCP concentration released into the digestive juices, as well as the formation of PCP complexes that interact with the digestive juices, may affect the absorption of PCP in intestinal cell models [28,45]. Related results suggest that steaming and boiling are better cooking methods, which do not significantly increase the bioavailability of PCP in food after cooking.

### 3.6. Effects of Food Matrices on the Bioavailability of PCP

**Figure 2:**
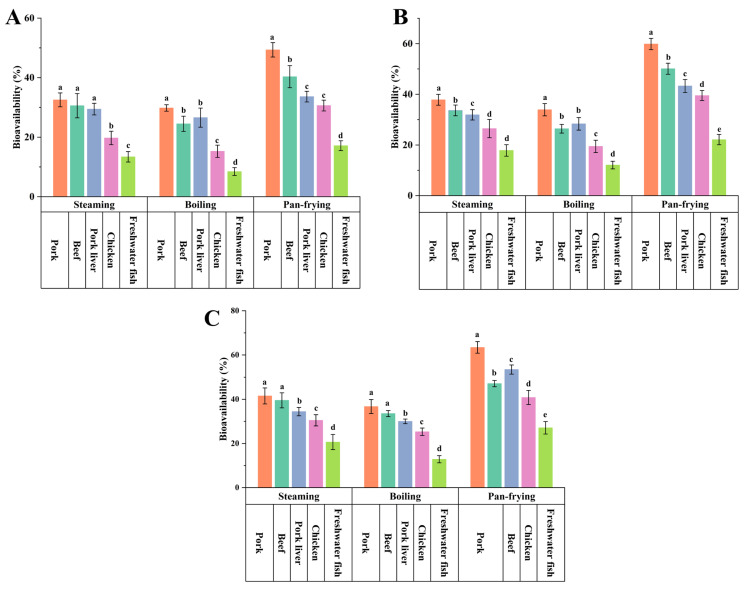
Effects of food matrices on the bioavailability of pentachlorophenol: (**A**) 100 μg/kg ww food samples; (**B**) 600 μg/kg ww food samples; (**C**) 1200 μg/kg ww food samples. Different food matrices with the mean values of different lower-case letters, treated by each cooking method, are statistically significant (*p* < 0.05).

Among the food samples with low, medium, and high PCP-spiked concentrations (Figure 2), the PCP bioavailability in pork samples with higher fat content was significantly higher than that of the other four food matrices after boiling, steaming, and pan-frying (*p* < 0.05), ranging from 29.81% to 63.41%. This was followed by beef samples, in which PCP bioavailability ranged from 24.49% to 53.43%. The lowest PCP bioavailability was found in freshwater fish samples (*p* < 0.05), ranging from 8.45% to 27.09%. The correlation results indicated that the fat content in the food matrix was an important factor affecting the bioavailability of PCP. In addition, other components in the food matrix, such as proteins and minerals, also affected the absorption of PCP and other POPs in the intestinal cells because the related nutrients competed with PCP for binding to transport proteins in the intestinal cells [28,46].

### 3.7. Dietary Exposure Assessment Based on Bioaccessibility and Bioavailability

The bioaccessibility and bioavailability of the five animal-derived foods were determined through cooking and in vitro digestion and absorption using a 100 μg/kg ww spiked concentration. The level of PCP exposure from the five animal-sourced foods was adjusted and evaluated for the general population, children (6–18 years old), and adults (18–70 years old) in the Guangdong Province, and the results are shown in Table 7, Table S6 and S7. PCP exposure levels, after correction for bioaccessibility and bioavailability, significantly decreased in the general population, as well as in juveniles and adults; the range was as follows: from 59.84 to 98.46% for low contamination, 49.56 to 97.20% for medium contamination, and 42.70 to 96.10% for high contamination. The calculation of PCP intake based on original PCP level in animal-derived food overestimated PCP exposure compared to those calculated based on bioavailable PCP. For the scenario of high contamination, the result of the PCP exposure risk assessment indicated that the estimated PCP intake based on the total PCP level in pork for local residents was close to the acceptable daily intake (ADI) of 3 μg/kg bw established by the United States National Research Council; this poses a potential health risk, especially for children [47]. However, the coupling of bioaccessibility or bioavailability information with risk assessment sharply reduced the dietary exposure risk of PCP via animal-derived foods. Compared with the dietary exposure evaluation, unlike the original risk analysis based on the total concentration in foods described in our previous report [1], the dietary exposure evaluation of bioaccessibility and bioavailability would greatly improve the accuracy of the exposure evaluation.

Although the combination of bioaccessibility and bioavailability of PCP obtained from in vitro models will provide an accurate risk assessment, it has some limitations that result in only approximations in comparison with the results of in vivo bioavailability; specifically, unclear potential modifiers of bioaccessibility and bioavailability result in a relatively poor replication, as well as the absence of intestinal flora which fails to simulate the complexity of gastrointestinal digestion [48,49]. Thus, future research should focus on determining the bioaccessibility and bioavailability from a large survey of animal-source samples; it should be conducted to identify the potential modifiers of bioaccessibility and bioavailability. In vivo−in vitro correlations between in vitro bioavailability and in vivo bioavailability of PCP should be established to further evaluate the efficiency of in vitro simulated models that introduce human intestinal microbiota for absorption.

## 4. Conclusions

This study mainly discussed the effect of different cooking methods and food matrices on the bioaccessibility and bioavailability of PCP using an in vitro digestive model. It evaluated the level of PCP exposure via foods of animal origin in the general population through the obtained bioaccessibility and bioavailability values. Cooking methods and food matrices significantly affected the bioaccessibility and bioavailability of PCP in animal-derived foods, with the bioaccessibility of PCP ranging from 21.82% to 90.36% and bioavailability from 12.86% to 63.41%. The pan-frying method using cooking oil was an important factor influencing the digestion and absorption of PCP in foods, with the bioaccessibility and bioavailability of pan-fried animal-derived foods ranging from 21.82% to 90.36% and 27.09% to 63.41%, respectively. Moreover, food matrices with higher fat content (such as pork and beef) were key factors facilitating the digestion and absorption of PCP in foods, contributing to the bioaccessibility (47.92–90.36%) and bioavailability (24.49–63.41%) of PCP. Therefore, it is advisable to consume foods with lower fat content and employ boiling or steaming for cooking food. The results of dietary exposure adjusted by bioaccessibility and bioavailability showed that, after considering bioaccessibility and bioavailability, the level of PCP exposure to the population via animal-derived foods was significantly reduced by 42.70–98.46%, with no potential health risk.

To the best of our knowledge, this is the first study to investigate the effects of the cooking process and food matrix on the in vitro bioaccessibility of PCP in food and its intestinal absorption using a Caco-2 model. The results suggest that incorporating bioaccessibility and bioavailability is crucial in the dietary exposure evaluations of food contaminants because the cooking process and food matrix regulate the release and metabolism of contaminants during gastrointestinal digestion; moreover, interactions between the cooking process and food matrix result in different absorption rates of PCP. Such findings highlight the need to consider bioaccessibility and bioavailability and their impact factors in PCP risk assessments. Thus, exposure risks could be overestimated when the bioaccessibility and bioavailability of PCP are not considered. We recommend introducing the bioaccessibility and bioavailability of PCP to adjust the dietary exposure level, ensuring more accurate exposure results and improving the accuracy of the data support for regulatory authorities to develop national food safety standard.

## Figures and Tables

**Table 1:** Condition of culinary treatment.

Culinary Treatment	Condition
Steaming	After the water boils, the sample is added and steamed for 5 min
Boiling	After 500 mL of distilled water boils, the sample is added and boiled for 5 min
Pan-frying	Sample is fried in 20 mL peanut oil at 180 °C for 3 min

**Table 2:** Background levels of pentachlorophenol in five animal origin foods.

Groups	Background Levels (μg/kg ww)
Pork	<LOQ
Beef	<LOQ
Pork liver	1.23 ± 0.16
Chicken	<LOQ
Freshwater fish	<LOQ

**Table 3:** Effects of pentachlorophenol-fortified samples (100 μg/kg ww) on the bioaccessibility and bioavailability of pentachlorophenol.

Group	Culinary Treatment	Fortification	Bioaccessibility (%)	Bioavailability (%)
Pork	Steaming	Fortified	60.52 ± 4.43 *	38.47 ± 2.41
		Naturally contaminated	57.55 ± 3.64	39.55 ± 3.14
	Boiling	Fortified	54.48 ± 2.47	32.69 ± 2.12
		Naturally contaminated	53.77 ± 4.46	33.77 ± 2.84
	Pan-frying	Fortified	85.66 ± 2.37	58.33 ± 2.41
		Naturally contaminated	86.74 ± 1.53	57.84 ± 3.32
Beef	Steaming	Fortified	55.49 ± 2.43	34.37 ± 3.12
		Naturally contaminated	56.77 ± 3.15	35.22 ± 2.64
	Boiling	Fortified	50.66 ± 2.84	28.38 ± 3.71
		Naturally contaminated	50.17 ± 2.11	29.52 ± 3.23
	Pan-frying	Fortified	80.36 ± 2.69 *	48.92 ± 3.71
		Naturally contaminated	78.22 ± 3.49	50.37 ± 4.03
Pork liver	Steaming	Fortified	52.17 ± 4.01	30.74 ± 2.52
		Naturally contaminated	49.37 ± 2.46	29.47 ± 3.10
	Boiling	Fortified	49.83 ± 2.61	26.54 ± 3.47
		Naturally contaminated	47.63 ± 3.23	27.67 ± 2.34
	Pan-frying	Fortified	73.46 ± 3.58	42.76 ± 2.97
		Naturally contaminated	74.84 ± 2.79	41.57 ± 2.46
Chicken	Steaming	Fortified	47.67 ± 3.18	24.32 ± 3.47
		Naturally contaminated	48.33 ± 2.73	23.69 ± 2.86
	Boiling	Fortified	41.51 ± 3.52	20.73 ± 2.11
		Naturally contaminated	39.41 ± 2.44	22.34 ± 1.96
	Pan-frying	Fortified	72.10 ± 3.29	37.62 ± 4.31
		Naturally contaminated	71.04 ± 2.91	39.13 ± 2.56
Freshwater fish	Steaming	Fortified	29.93 ± 3.37	16.62 ± 2.62
		Naturally contaminated	30.45 ± 1.74	17.97 ± 3.13
	Boiling	Fortified	24.94 ± 3.37	10.84 ± 2.85
		Naturally contaminated	22.62 ± 3.83	11.56 ± 1.43
	Pan-frying	Fortified	67.64 ± 2.28	20.74 ± 2.18
		Naturally contaminated	69.16 ± 1.57	22.04 ± 2.63

In the same column of each food matrix treated by the same culinary method, the mean values with an asterisk were statistically significant (*p* < 0.05), with the corresponding results of the food matrix containing background PCP.

**Table 4:** Effects of culinary treatments on the bioaccessibility of pentachlorophenol in five food matrices (100 μg/kg ww).

Group	Culinary Treatment	Bioaccessibility (%)		
		Oral Cavity	Stomach	Small Intestine
Pork	Steaming	12.51 ± 1.56 ^aA^	51.05 ± 2.88 ^aB^	58.86 ± 2.43 ^aC^
	Boiling	11.63 ± 2.08 ^aA^	46.39 ± 2.24 ^bB^	50.61 ± 2.17 ^bB^
	Pan-frying	16.80 ± 1.17 ^bA^	70.64 ± 3.59 ^cB^	81.37 ± 2.16 ^cC^
Beef	Steaming	10.35 ± 1.77 ^abA^	42.51 ± 2.29 ^aB^	50.39 ± 2.08 ^aC^
	Boiling	8.90 ± 2.06 ^aA^	38.74 ± 3.06 ^aB^	47.92 ± 1.46 ^aC^
	Pan-frying	13.83 ± 1.44 ^bA^	64.50 ± 2.31 ^bB^	72.09 ± 3.54 ^bC^
Pork liver	Steaming	8.52 ± 2.01 ^abA^	37.89 ± 3.02 ^aB^	48.76 ± 2.49 ^aC^
	Boiling	6.41 ± 1.74 ^aA^	33.54 ± 2.62 ^bB^	41.54 ± 2.18 ^bC^
	Pan-frying	10.08 ± 2.52 ^bA^	56.43 ± 3.08 ^cB^	69.11 ± 3.02 ^cC^
Chicken	Steaming	7.09 ± 2.18 ^abA^	30.48 ± 1.96 ^aB^	40.71 ± 3.92 ^aC^
	Boiling	6.73 ± 1.50 ^aA^	24.53 ± 2.27 ^bB^	31.32 ± 2.48 ^bC^
	Pan-frying	9.89 ± 1.63 ^bA^	51.08 ± 1.62 ^cB^	63.43 ± 3.27 ^cC^
Freshwater fish	Steaming	7.72 ± 1.46 ^abA^	18.02 ± 2.51 ^aB^	27.31 ± 3.62 ^aC^
	Boiling	5.62 ± 1.84 ^aA^	14.14 ± 2.46 ^aB^	21.82 ± 2.62 ^bC^
	Pan-frying	8.86 ± 2.04 ^bA^	45.72 ±3.14 ^bB^	60.27 ± 2.25 ^cC^

In the same column of each food matrix, the mean values of different lowercase letters are statistically significant (*p* < 0.05). The average values of different capital letters in the same line are statistically significant (*p* < 0.05).

**Table 5:** Effects of culinary treatments on the bioaccessibility of pentachlorophenol in five food matrices (600 μg/kg ww).

Group	Culinary Treatment	Bioaccessibility (%)		
		Oral Cavity	Stomach	Small Intestine
Pork	Steaming	20.32 ± 1.83 ^aA^	53.92 ± 1.96 ^aB^	62.83 ± 3.52 ^aC^
	Boiling	13.83 ± 1.27 ^bA^	46.49 ± 1.53 ^bB^	52.91 ± 3.19 ^bB^
	Pan-frying	27.16 ± 3.19 ^cA^	78.17 ± 3.12 ^cB^	84.27 ± 4.14 ^cB^
Beef	Steaming	14.34 ± 2.51 ^aA^	50.84 ± 2.09 ^aB^	58.33 ± 2.48 ^aC^
	Boiling	10.42 ± 2.16 ^aA^	46.35 ± 1.55 ^bB^	53.73 ± 2.16 ^bC^
	Pan-frying	23.61 ± 3.52 ^bA^	68.03 ± 2.41 ^cB^	79.62 ± 1.74 ^cC^
Pork liver	Steaming	11.63 ± 1.64 ^aA^	44.83 ± 3.14 ^aB^	54.41 ± 2.66 ^aC^
	Boiling	10.11 ± 1.47 ^aA^	47.41 ± 2.61 ^aB^	52.18 ± 2.07 ^aB^
	Pan-frying	18.19 ± 2.67 ^bA^	61.53 ± 1.74 ^bB^	71.25 ± 3.12 ^bC^
Chicken	Steaming	8.23 ± 0.96 ^aA^	36.56 ± 2.09 ^aB^	48.02 ± 2.33 ^aC^
	Boiling	8.05 ± 1.23 ^aA^	30.74 ± 2.08 ^bB^	40.09 ± 2.62 ^bC^
	Pan-frying	14.63 ± 2.83 ^bA^	63.58 ± 2.14 ^cB^	71.73 ± 2.76 ^cC^
Freshwater fish	Steaming	7.03 ± 1.50 ^abA^	22.61 ± 3.46 ^aB^	30.45 ± 1.86 ^aC^
	Boiling	4.93 ± 0.86 ^aA^	19.05 ± 2.78 ^aB^	23.19 ± 2.54 ^bB^
	Pan-frying	9.19 ± 1.26 ^bA^	53.46 ± 2.13 ^bB^	69.13 ± 2.47 ^cC^

In the same column of each food matrix, the mean values of different lowercase letters are statistically significant (*p* < 0.05). The average values of different capital letters in the same line are statistically significant (*p* < 0.05).

**Table 6:** Effects of culinary treatments on the bioaccessibility of pentachlorophenol in five food matrices (1200 μg/kg ww).

Group	Culinary Treatment	Bioaccessibility (%)		
		Oral Cavity	Stomach	Small Intestine
Pork	Steaming	23.41 ± 1.86 ^aA^	61.95 ± 3.48 ^aB^	70.53 ± 4.34 ^aC^
	Boiling	17.05 ± 0.96 ^bA^	53.46 ± 2.47 ^bB^	62.39 ± 4.62 ^bC^
	Pan-frying	35.03 ± 1.85 ^cA^	81.57 ± 1.62 ^cB^	90.36 ± 3.38 ^cC^
Beef	Steaming	19.39 ± 2.21 ^aA^	58.91 ± 4.11 ^aB^	66.41 ± 2.34 ^aC^
	Boiling	15.18 ± 1.53 ^bA^	50.63 ± 3.18 ^bB^	60.16 ± 3.21 ^bC^
	Pan-frying	28.86 ± 3.16 ^cA^	73.89 ± 3.24 ^cB^	83.63 ± 4.01 ^cC^
Pork liver	Steaming	15.71 ± 2.63 ^aA^	48.32 ± 2.54 ^aB^	60.53 ± 4.62 ^aC^
	Boiling	12.08 ± 1.74 ^aA^	44.69 ± 1.86 ^aB^	56.32 ± 1.35 ^bC^
	Pan-frying	22.12 ± 3.18 ^bA^	70.46 ± 2.06 ^bB^	78.07 ± 2.37 ^cC^
Chicken	Steaming	11.80 ± 2.76 ^aA^	44.56 ± 3.36 ^aB^	57.06 ± 3.14 ^aC^
	Boiling	10.03 ± 1.76 ^aA^	35.09 ± 4.02 ^bB^	49.24 ± 3.39 ^bC^
	Pan-frying	18.09 ± 1.54 ^bA^	68.42 ± 3.83 ^cB^	75.52 ±1.74 ^cC^
Freshwater fish	Steaming	9.26 ± 1.86 ^aA^	29.84 ± 2.65 ^aB^	36.24 ± 2.05 ^aC^
	Boiling	8.52 ± 0.69 ^bA^	24.26 ± 2.78 ^bB^	30.36 ± 3.47 ^bC^
	Pan-frying	12.26 ± 1.41 ^cA^	62.48 ± 1.71 ^cB^	72.14 ± 2.73 ^cC^

In the same column of each food matrix, the mean values of different lowercase letters are statistically significant (*p* < 0.05). The average values of different capital letters in the same line are statistically significant (*p* < 0.05).

**Table 7:** Bioaccessibility and bioavailability were adjusted based on estimates of the daily intake of pentachlorophenol (scenario of low contamination: 100 μg/kg bw) in terms of the five food groups consumed by the general population, children (age 6–17 years) and adults (age 18–70 years) at average consumption levels.

Group	Culinary Treatments	Bioaccessibility (%)	Bioavailability (%)	Estimated Daily Intakes (μg/kg bw)				
				General Population ^a^	Boys ^b^	Girls ^c^	Male Adults ^d^	Female Adults ^e^
Pork	Conventional assumption	100	100	0.171	0.214	0.215	0.164	0.129
	Steaming	58.86	32.54	0.033	0.041	0.041	0.031	0.025
	Boiling	50.61	29.81	0.026	0.032	0.032	0.025	0.019
	Pan-frying	81.37	49.34	0.069	0.086	0.086	0.066	0.052
Beef	Conventional assumption	100	100	0.017	0.016	0.012	0.013	0.012
	Steaming	50.39	30.58	0.003	0.002	0.002	0.002	0.002
	Boiling	47.92	24.49	0.002	0.002	0.001	0.002	0.001
	Pan-frying	72.09	40.32	0.005	0.005	0.004	0.004	0.004
Pork liver	Conventional assumption	100	100	0.012	0.010	0.011	0.012	0.011
	Steaming	48.76	28.43	0.002	0.001	0.001	0.002	0.001
	Boiling	41.54	26.57	0.001	0.001	0.001	0.001	0.001
	Pan-frying	69.11	33.63	0.003	0.002	0.002	0.003	0.003
Chicken	Conventional assumption	100	100	0.065	0.075	0.065	0.062	0.067
	Steaming	40.71	19.72	0.005	0.006	0.005	0.005	0.005
	Boiling	31.32	15.27	0.003	0.004	0.003	0.003	0.003
	Pan-frying	63.43	30.63	0.013	0.014	0.013	0.012	0.013
Freshwater fish	Conventional assumption	100	100	0.092	0.076	0.105	0.098	0.099
	Steaming	27.31	13.40	0.003	0.003	0.004	0.004	0.004
	Boiling	21.82	8.45	0.002	0.001	0.002	0.002	0.002
	Pan-frying	60.27	17.14	0.010	0.008	0.011	0.010	0.010

Average body weight: (a) general population: 60.8 kg; (b) boys: 46.5 kg; (c) girls: 41.1 kg; (d) male adults: 64.5 kg; (e) female adults: 54.6 kg.

## Data Availability

The original contributions presented in the study are included in the article/Supplementary Material, further inquiries can be directed to the corresponding authors.

## Supplementary Materials

The following supporting information can be downloaded at: https://www.mdpi.com/article/10.3390/foods13081254/s1, Figure S1. Results of CCK8 assay of five food matrices at 1200 μg/kg ww; Figure S2. Results of validation characteristics for Caco-2 monolayer cell model: (a) image of Caco-2 cell model; (b) transmembrane resistance value of the Caco-2 cell model; (c) AKP activity of Caco-2 cell model; (d) Lucifer yellow permeability coefficient; Table S1. Chemical composition of digestive juice (per liter); Table S2. Gradient elution conditions for liquid chromatography; Table S3. The results of recovery test (n = 5) of PCP in five food matrices; Table S4. Average daily intake (g) of five animal-derived foods of different populations; Table S5. Effect of food matrix on bioaccessibility of pentachlorophenol; Table S6. Bioaccessibility and bioavailability were adjusted based on estimates of the daily intake of pentachlorophenol (scenario of medium contamination: 600 μg/kg bw) in terms of the five food groups consumed by the general population, children (age 6–17 years) and adults (age 18–70 years) at average consumption levels; Table S7. Bioaccessibility and bioavailability were adjusted based on estimates of the daily intake of pentachlorophenol (scenario of high contamination: 1200 μg/kg bw) in terms of the five food groups consumed by the general population, children (age 6–17 years) and adults (age 18–70 years) at average consumption levels.
