# Supplementary material for: The Bioaccessibility and Bioavailability of Pentachlorophenol in Five Animal-Derived Foods Measured by Simulated Gastrointestinal Digestion
Source: Foods. 2024 Apr 19;13(8):1254. doi: 10.3390/foods13081254 (PMC11049475; doi:10.3390/foods13081254)
Supplement: Supplementary file 1 [file foods-13-01254-s001.zip › foods-2935410-supplementary.pdf]

<u>**Supplemental Materials**</u>

The bioaccessibility and bioavailability of pentachlorophenol in five animal-derived foods measured by simulated gastrointestinal digestion

Quan Zhou [1], Hui-Ming Chen [2], Liang-Liang Li [2], Yong-Ning Wu [3], Xing-Fen Yang [2], Ai-Min Jiang [1,] *, Wei-Liang Wu [2,] *

[1] The National Center for Precision Machining and Safety of Livestock and Poultry Products Joint Engineering Research Center, College of Food Science, South China Agricultural University, Guangzhou 510642, P. R. China

[2] Food Safety and Health Research Center, Guangdong Provincial Key Laboratory of Tropical Disease Research, Guangdong-Hongkong-Macao Joint Laboratory for Contaminants Exposure and Health, School of Public Health, Southern Medical University, Guangzhou 510515, P. R. China

[3] National Center for Food Safety Risk Assessment, Key Laboratory of Food Safety Risk Assessment, Ministry of Health, Beijing 100021, P. R. China.

Correspondence: Wei-Liang Wu: wu1108@smu.edu.cn; Ai-Min Jiang: amjiang@sacu.edu.cn

Figure Captions

Figure S1. Results of CCK8 assay of five food matrices at 1200 μg/kg ww.

Figure S2. Results of validation characteristics for Caco-2 monolayer cell model.

Tables

Table S1. Physical-Chemical properties of 8 chlorophenols.

Table S2. Gradient elution conditions for liquid chromatography.

Table S3. The results of recovery test (n = 5) of PCP in five food matrices.

Table S4. Average daily intake (g) of five animal-derived foods of different populations.

Table S5. Effect of food matrix on bioaccessibility of pentachlorophenol.

Table S6. Bioaccessibility and bioavailability were adjusted based on estimates of the daily intake of pentachlorophenol (scenario of medium contamination: 600 μg/kg bw) in terms of the five food groups consumed by the general population, children (age 6–17 years) and adults (age 18–70 years) at average consumption levels.

Table S7. Bioaccessibility and bioavailability were adjusted based on estimates of the daily intake of pentachlorophenol (scenario of high contamination: 1200 μg/kg bw) in terms of the five food groups consumed by the general population, children (age 6–17 years) and adults (age 18–70 years) at average consumption levels.

Consumption and body weight data [1]

The data of daily consumption of animal-derived foods and body weight of local residents were obtained from the National Nutrition and Health Survey (NAHS) conducted by the

Guangdong Provincial Center for Disease Control and Prevention in 2012. A total of 3780 households were selected from 9 counties/districts in Guangdong Province, out of which 2112 households were surveyed, including 1297 urban households and 815 rural households. A comprehensive survey was conducted on a total of 5,179 residents aged between 6 to 70 years over a period of three consecutive days, capturing their food and nutrient intake patterns, encompassing average consumption levels of five animal-derived foods.

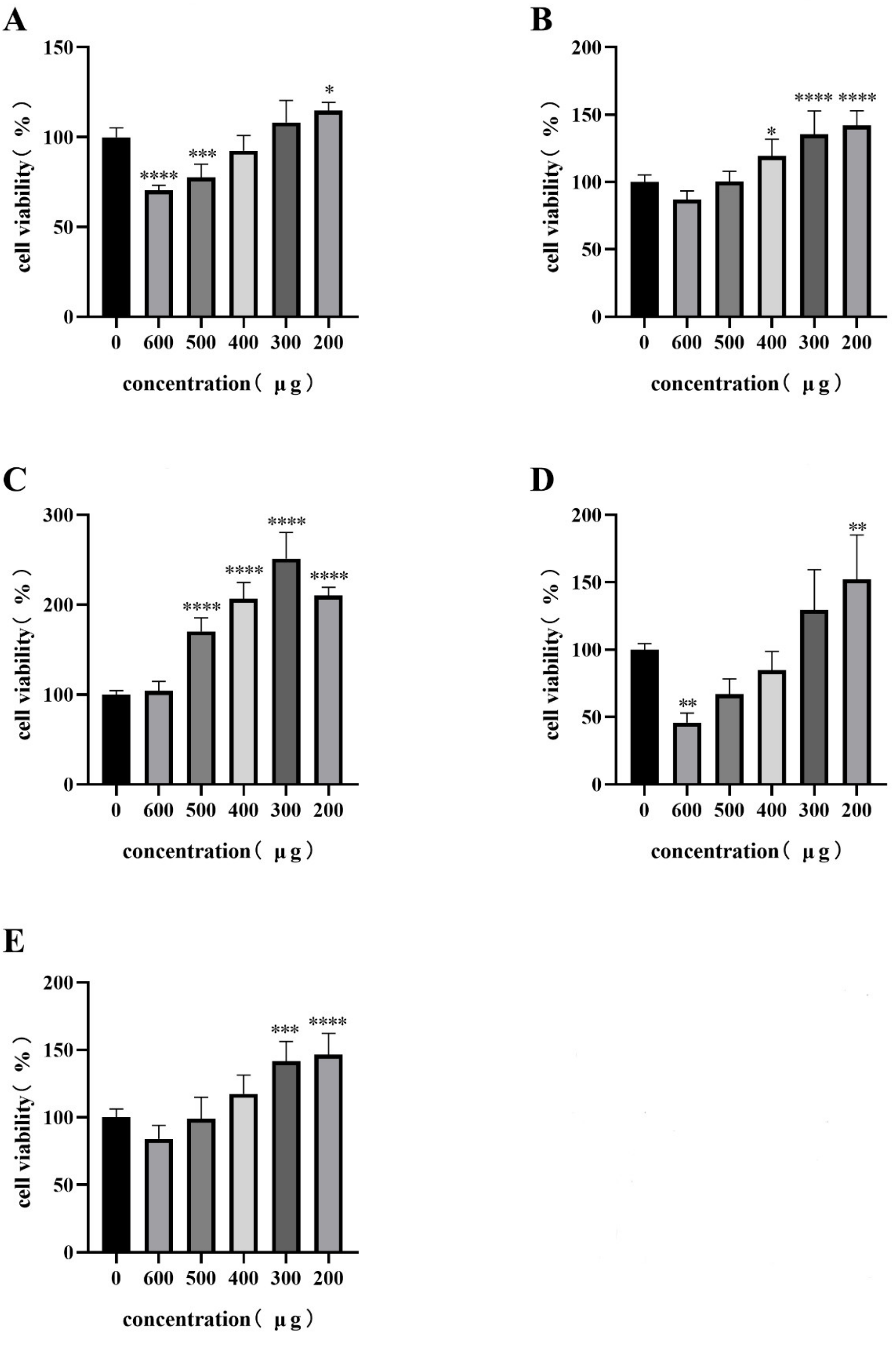

Figure S1. Results of CCK8 assay of five food matrices at 1200 μg/kg ww: (a) pork; (b) beef; (c) pork liver; (d) chicken; (e) freshwater fish. * $p < 0.05$ vs control group; ** $p < 0.01$ vs control group; *** $p < 0.001$ vs control group; **** $p < 0.0001$ vs control group;

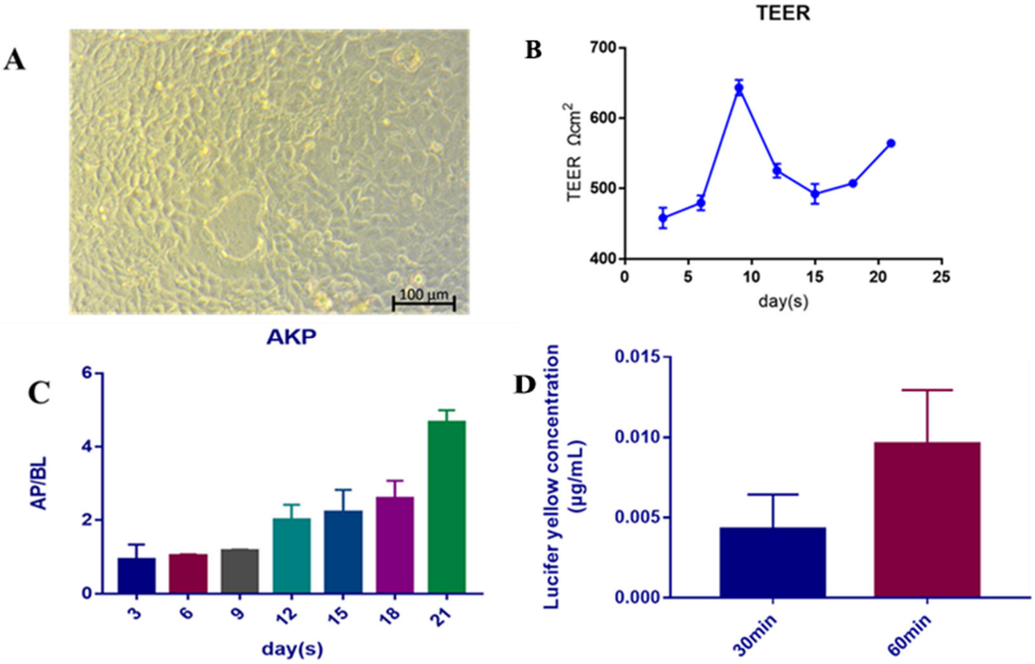

Figure S2. Results of validation characteristics for Caco-2 monolayer cell model: (a) image of Caco-2 cell model; (b) transmembrane resistance value of the Caco-2 cell model; (c) AKP activity of Caco-2 cell model; (d) Lucifer yellow permeability coefficient.

Table S1. Chemical composition of digestive juice (per liter)

Oral phase	Gastric juice	Duodenal juice	Bile juice
0.9 g KCl	2.75 g NaCl	7.01 g NaCl	5.26 g NaCl
0.2 g KSCN	0.27 g NaH_2PO_4	3.39 g $NaHCO_3$	5.79 g $NaHCO_3$
0.9 g NaH_2PO_4	0.82 g KCl	0.08 g KH_2PO_4	0.38 g KCl
0.57 g Na_2SO_4	0.4 g $CaCl_2{\cdot}2H_2O$	0.56 g KCl	0.25 g urea
0.3 g NaCl	0.31 g NH_4Cl	0.05 g $MgCl_2$	0.22 g $CaCl_2{\cdot}2H_2O$
1.7 g $NaHCO_3$	0.65 g glucose	0.1 g urea	1.8 g BSA
0.2 g urea	0.02 g glucuronic acid	0.2 g $CaCl_2{\cdot}2H_2O$	30 g bile
290 mg α-amylase	0.085 g urea	1 g BSA	pH 6.5
15 mg uric acid	0.33 g glucosamine hydrochloride	9 g pancreatin	
25 mg mucin	1 g BSA	1.5 g lipase	
pH 6.5	2.5 g pepsin	pH 7.5	
	3 g mucin		
	pH 2.0		

Table S2. Gradient elution conditions for liquid chromatography.

Time (min)	Elution A (%)	Elution B (%)
0.00	40	60
1.00	100	0
7.00	100	0
7.50	40	60
12.00	40	60

Table S3. The results of recovery test (*n* = 5) of PCP in five food matrices.

Group	Recoveries (%)		
	100 μg/kg	600 μg/kg	1200 μg/kg
Pork	98.6 ± 3.38	102.6 ± 3.63	103.3 ± 4.26
Beef	96.9 ± 2.96	100.7 ± 2.84	106.8 ± 3.71
Pork liver	96.5 ± 3.54	98.2 ± 3.82	100.6 ± 3.22
Chicken	97.1 ± 2.89	97.1 ± 4.11	99.2 ± 4.05
Freshwater fish	97.8 ± 3.72	99.5 ± 3.69	100.8 ± 2.75

Table S4. Average daily intake (g) of five animal-derived foods of different populations.

Food groups	General population	Boys	Girls	Male adults	Female adults
Pork	104.2	99.7	88.5	106.0	70.4
Beef	10.3	7.4	5.1	8.6	6.7
Pork liver	7.3	4.5	4.4	8.0	5.9
Chicken	39.3	34.7	26.9	40.1	36.8
Freshwater fish	56.2	35.2	43.0	63.0	54.1

Table S5. Effect of food matrix on bioaccessibility of pentachlorophenol.

Concentration (μg/kg ww)	Culinary treatment	Group	Bioaccessibility (%)		
			Oral cavity	Stomach	Small intestine
100	Steaming	Pork	12.51 ± 1.56^{a}	51.05 ± 2.88^{a}	58.86 ± 2.43^{a}
		Beef	10.35 ± 1.77^{ab}	42.51 ± 2.29^{b}	50.39 ± 2.08^{b}
		Pork liver	8.52 ± 2.01^{b}	37.89 ± 3.02^{c}	48.76 ± 2.49^{b}
		Chicken	7.09 ± 2.18^{b}	30.48 ± 1.96^{d}	40.71 ± 3.92^{c}
		Freshwater fish	7.72 ± 1.46^{b}	18.02 ± 2.51^{e}	27.31 ± 3.62^{d}
	Boiling	Pork	11.63 ± 2.08^{a}	46.39 ± 2.24^{a}	50.61 ± 2.17^{a}
		Beef	8.90 ± 2.06^{b}	38.74 ± 3.06^{b}	47.92 ± 1.46^{a}
		Pork liver	6.41 ± 1.74^{bc}	33.54 ± 2.62^{c}	41.54 ± 2.18^{b}
		Chicken	6.73 ± 1.50^{bc}	24.53 ± 2.27^{d}	31.32 ± 2.48^{c}
		Freshwater fish	5.62 ± 1.84^{c}	14.14 ± 2.46^{e}	21.82 ± 2.62^{d}
	Pan-frying	Pork	16.80 ± 1.17^{a}	70.64 ± 3.59^{a}	81.37 ± 2.16^{a}
		Beef	13.83 ± 1.44^{b}	64.50 ± 2.31^{b}	72.09 ± 3.54^{b}
		Pork liver	10.08 ± 2.52^{c}	56.43 ± 3.08^{c}	69.11 ± 3.02^{c}
		Chicken	9.89 ± 1.63^{c}	51.08 ± 1.62^{d}	63.43 ± 3.27^{d}
		Freshwater fish	8.86 ± 2.04^{c}	45.72 ±3.14^{e}	60.27 ± 2.25^{d}
600	Steaming	Pork	20.32 ± 1.83^{a}	53.92 ± 1.96^{a}	62.83 ± 3.52^{a}
		Beef	14.34 ± 2.51^{b}	50.84 ± 2.09^{a}	58.33 ± 2.48^{b}
		Pork liver	11.63 ± 1.64^{c}	44.83 ± 3.14^{b}	54.41 ± 2.66^{c}
		Chicken	8.23 ± 0.96^{d}	36.56 ± 2.09^{c}	48.02 ± 2.33^{d}
		Freshwater fish	7.03 ± 1.50^{d}	22.61 ± 3.46^{d}	30.45 ± 1.86^{e}
	Boiling	Pork	13.83 ± 1.27^{a}	46.49 ± 1.53^{a}	52.91 ± 3.19^{a}
		Beef	10.42 ± 2.16^{b}	46.35 ± 1.55^{a}	53.73 ± 2.16^{a}
		Pork liver	10.11 ± 1.47^{b}	47.41 ± 2.61^{a}	52.18 ± 2.07^{a}
		Chicken	8.05 ± 1.23^{b}	30.74 ± 2.08^{b}	40.09 ± 2.62^{b}
		Freshwater fish	4.93 ± 0.86^{c}	19.05 ± 2.78^{c}	23.19 ± 2.54^{c}
	Pan-frying	Pork	27.16 ± 3.19^{a}	78.17 ± 3.12^{a}	84.27 ± 4.14^{a}
		Beef	23.61 ± 3.52^{b}	68.03 ± 2.41^{b}	79.62 ± 1.74^{b}
		Pork liver	18.19 ± 2.67^{c}	61.53 ± 1.74^{c}	71.25 ± 3.12^{c}
		Chicken	14.63 ± 2.83^{d}	63.58 ± 2.14^{c}	71.73 ± 2.76^{c}
		Freshwater fish	9.19 ± 1.26^{e}	53.46 ± 2.13^{d}	69.13 ± 2.47^{c}
1200	Steaming	Pork	23.41 ± 1.86^{a}	61.95 ± 3.48^{a}	70.53 ± 4.34^{a}
		Beef	19.39 ± 2.21^{b}	58.91 ± 4.11^{a}	66.41 ± 2.34^{b}
		Pork liver	15.71 ± 2.63^{c}	48.32 ± 2.54^{b}	60.53 ± 4.62^{c}
		Chicken	11.80 ± 2.76^{d}	44.56 ± 3.36^{b}	57.06 ± 3.14^{c}
		Freshwater fish	9.26 ± 1.86^{d}	29.84 ± 2.65^{d}	36.24 ± 2.05^{d}
	Boiling	Pork	17.05 ± 0.96^{a}	53.46 ± 2.47^{a}	62.39 ± 4.62^{a}
		Beef	15.18 ± 1.53^{a}	50.63 ± 3.18^{a}	60.16 ± 2.21^{a}
		Pork liver	12.08 ± 1.74^{b}	44.69 ± 1.86^{b}	56.32 ± 1.35^{b}
		Chicken	10.03 ± 1.76^{bc}	35.09 ± 4.02^{c}	49.24 ± 3.39^{c}
		Freshwater fish	8.52 ± 0.69^{c}	24.26 ± 2.78^{d}	30.36 ± 3.47^{d}
	Pan-frying	Pork	35.03 ± 1.85^{a}	81.57 ± 1.62^{a}	90.36 ± 3.38^{a}
		Beef	28.86 ± 3.16^{b}	73.89 ± 3.24^{b}	83.63 ± 4.01^{b}
		Pork liver	22.12 ± 3.18^{c}	70.46 ± 2.06^{bc}	78.07 ± 2.37^{c}
		Chicken	18.09 ± 1.54^{d}	68.42 ± 3.83^{c}	75.52 ±1.74^{cd}
		Freshwater fish	12.26 ± 1.41^{e}	62.48 ± 1.71^{d}	72.14 ± 2.73^{d}

In the same column of each cooking method, the average values of food matrices with different lowercase letters have statistically significant differences ($p < 0.05$).

Table S6. Bioaccessibility and bioavailability were adjusted based on estimates of the daily intake of pentachlorophenol (scenario of medium contamination: 600 μg/kg bw) in terms of the five food groups consumed by the general population, children (age 6–17 years) and adults (age 18–70 years) at average consumption levels.

Group	Culinary treatments	Bioaccessibility （%）	Bioavailability （%）	Estimated daily intakes（μg/kg bw）				
				**General population**	**Boys**	**Girls**	**Male adults**	**Female adults**
Pork	Conventional assumption	100	100	1.028	1.286	1.292	0.986	0.774
	Steaming	62.83	37.84	0.244	0.306	0.307	0.234	0.184
	Boiling	52.91	33.92	0.184	0.231	0.232	0.177	0.139
	Pan-frying	84.27	59.86	0.519	0.649	0.652	0.497	0.390
Beef	Conventional assumption	100	100	0.102	0.096	0.075	0.080	0.074
	Steaming	58.33	33.64	0.020	0.019	0.015	0.016	0.014
	Boiling	53.73	26.42	0.014	0.014	0.011	0.011	0.010
	Pan-frying	79.62	50.1	0.041	0.038	0.030	0.032	0.029
Pork liver	Conventional assumption	100	100	0.072	0.058	0.066	0.074	0.065
	Steaming	54.41	31.92	0.013	0.010	0.011	0.013	0.011
	Boiling	52.18	28.36	0.011	0.009	0.010	0.011	0.010
	Pan-frying	71.25	43.29	0.022	0.018	0.020	0.023	0.020
Chicken	Conventional assumption	100	100	0.388	0.448	0.393	0.373	0.404
	Steaming	48.02	26.47	0.049	0.057	0.050	0.047	0.051
	Boiling	40.09	19.05	0.030	0.034	0.030	0.028	0.031
	Pan-frying	71.73	39.51	0.110	0.127	0.111	0.106	0.114
Freshwater fish	Conventional assumption	100	100	0.555	0.454	0.628	0.586	0.595
	Steaming	30.45	17.84	0.030	0.025	0.034	0.032	0.032
	Boiling	23.19	12.08	0.016	0.013	0.018	0.016	0.017
	Pan-frying	69.13	22.11	0.085	0.069	0.096	0.090	0.091

Table S7. Bioaccessibility and bioavailability were adjusted based on estimates of the daily intake of pentachlorophenol (scenario of high contamination: 1200 μg/kg bw) in terms of the five food groups consumed by the general population, children (age 6–17 years) and adults (age 18–70 years) at average consumption levels.

Group	Culinary treatments	Bioaccessibility (%)	Bioavailability (%)	Estimated daily intakes (μg/kg bw)				
				General population	Boys	Girls	Male adults	Female adults
Pork	Conventional assumption	100	100	2.057	2.573	2.584	1.972	1.547
	Steaming	70.53	41.53	0.603	0.754	0.757	0.578	0.453
	Boiling	62.39	36.72	0.471	0.589	0.592	0.452	0.354
	Pan-frying	90.36	63.41	1.179	0.432	0.434	1.130	0.886
Beef	Conventional assumption	100	100	0.203	0.191	0.149	0.160	0.147
	Steaming	66.41	39.53	0.053	0.050	0.039	0.042	0.039
	Boiling	60.16	33.54	0.041	0.039	0.030	0.032	0.030
	Pan-frying	83.63	53.43	0.091	0.022	0.017	0.071	0.066
Pork liver	Conventional assumption	100	100	0.144	0.116	0.128	0.149	0.130
	Steaming	60.53	34.43	0.030	0.024	0.027	0.031	0.027
	Boiling	56.32	30.01	0.024	0.020	0.022	0.025	0.022
	Pan-frying	78.07	47.11	0.053	0.009	0.010	0.055	0.048
Chicken	Conventional assumption	100	100	0.776	0.895	0.785	0.746	0.809
	Steaming	57.06	30.48	0.135	0.156	0.137	0.130	0.141
	Boiling	49.24	25.31	0.097	0.112	0.098	0.093	0.101
	Pan-frying	75.52	40.83	0.239	0.048	0.042	0.230	0.249
Freshwater fish	Conventional assumption	100	100	1.109	0.908	1.255	1.172	1.189
	Steaming	36.24	20.64	0.083	0.068	0.094	0.088	0.089
	Boiling	30.36	12.86	0.043	0.035	0.049	0.046	0.046
	Pan-frying	72.14	27.09	0.217	0.013	0.018	0.229	0.232

Reference:

1. Zhang, Y.H.; Ma, W.J. Analysis for ten-years changes of dietary nutrition and health status of Guangdong residents, 1st ed.; Zhijian Publishing House: Beijing, China, 2016; pp. 102–115. (in Chinese)
